# Urogenital fascia anatomy study in the inguinal region of 10 formalin-fixed cadavers: new understanding for laparoscopic inguinal hernia repair

**DOI:** 10.1186/s12893-021-01287-z

**Published:** 2021-06-17

**Authors:** Yi Li, Changfu Qin, Likun Yan, Cong Tong, Jian Qiu, Yamin Zhao, Yang Xiao, Xiaoqiang Wang

**Affiliations:** 1 First Department of General Surgery, Shaanxi Provincial People’s Hospital, Xi’an, Shaanxi, 710000 China; 2 Department of Hernia and Abdominal Wall Surgery, Beijing Chaoyang Hospital, Capital Medical University, Beijing, 100043 China

**Keywords:** Urogenital fascia, Renal fascia, Preperitoneal fascia, Extraperitoneal fascia, Transversalis fascia, Presacral fascia

## Abstract

**Purpose:**

To investigate the urogenital fascia (UGF) anatomy in the inguinal region, to provide anatomical guidance for laparoscopic inguinal hernia repair (LIHR).

**Methods:**

The anatomy was performed on 10 formalin-fixed cadavers. The peritoneum and its deeper fascial tissues were carefully dissected.

**Results:**

The UGF’s bilateral superficial layer extended and ended in front of the abdominal aorta. At the posterior axillary line, the superficial layer medially reversed, with extension represented the UGF's deep layer. The UGF's bilateral deep layer medially extended beside the vertebral body and then continued with the transversalis fascia. The ureters, genital vessels, and superior hypogastric plexus moved between both layers. The vas deferens and spermatic vessels, ensheathed by both layers, moved through the deep inguinal ring. From the deep inguinal ring to the midline, the superficial layer extended to the urinary bladder’s posterior wall, whereas the deep layer extended to its anterior wall. Both layers ensheathed the urinary bladder and extended along the medial umbilical ligament to the umbilicus and in the sacral promontory, extended along the sacrum, forming the presacral fascia. The superficial layer formed the rectosacral fascia at S4 sacral vertebra, and the deep layer extended to the pelvic diaphragm, terminating at the levator ani muscle.

**Conclusion:**

The UGF ensheaths the kidneys, ureters, vas deferens, genital vessels, superior hypogastric plexus, seminal vesicles, prostate, and urinary bladder. This knowledge of the UGF’s anatomy in the inguinal region will help find correct LIHR targets and reduce bleeding and other complications.

## Introduction

It is difficult to distinguish between the retroperitoneal fascia and the fascia surrounding the pelvis. The same fascia is called by different names in different parts of the body, and the same name can be used for two different fasciae. The concept of the urogenital fascia (UGF) is not novel in the anatomical field. Diarra et al. [1] and Muntean et al. [2] proposed the concept of the UGF. Yang et al. [3] proposed the concept of a urogenital-hypogastric sheath. Both concepts consider the renal fascia as the core and define the renal fascia and its extensions as the UGF or the urogenital-hypogastric sheath. However, the boundaries and adjacent relationships between the UGF and the urogenital-hypogastric sheath remain unclear. During laparoscopic inguinal hernia repair (LIHR), surgeons not only focus on the anatomy of the membrane but also propose some related fasciae, such as preperitoneal, extraperitoneal, and umbilico–prevesical fasciae (UPF). However, their interrelationship remains unclear. Controversy also surrounds the stratification of the transversalis fascia [4, 5]. Currently, no studies have focused on the relationship among the UGF, UPF, preperitoneal fascia, and extraperitoneal fascia. These different definitions for the same fascia may interfere with our understanding of the inguinal membrane and even affect LIHR results. Therefore, understanding the anatomy of the UGF is required to distinguish relevant fasciae, unify the definitions of relevant fasciae, and provide a perfect anatomical basis for LIHR. This study explored the anatomical characteristics of the UGF and its clinical application value in surgery in the inguinal region.

## Subjects and methods

### Subjects

The entire study was carried out in strict line with protocols approved by the Biomedical Ethics Committee of Xi’an Jiaotong University (Ethics Permit Number: 2014-0303). The study was based on the dissection of 10 formalin-fixed cadavers (9 males and 1 female) provided by the Department of Anthropotomy and Histo-Embryology of Xi'an Jiaotong University Health Science Center, using one set of anatomic devices and one camera. Written informed consent was obtained from the immediate family members of the deceased for educational and scientific research purposes. The format of the informed consent form is in line with the guidelines of the China Organ Donation Administrative Center.

### Methods

In each cadaver, the peritoneum was horizontally incised at 5 cm from the deep inguinal ring in the anterior abdominal wall, lateral to the anterior superior iliac, and medial to the medial umbilical fold. First, the peritoneum and its deeper fascial tissues were carefully dissected. Next, the vas deferens and spermatic vessels in male cadavers were continuously separated from the sigmoid mesocolon root after exposing the deep inguinal ring. Internally and inferiorly, the space between the urinary bladder and pubic ramus was separated, exposing the pubic symphysis. After the anatomy of the inguinal hernia repair area was determined, the peritoneum at the bilateral paracolic gutter was longitudinally incised. Then, the ascending and descending colons and the sigmoid colon were medially separated from the sigmoid mesocolon root. Medially, the colon was lifted, exposing the retroperitoneal space. The presacral space was entered from the surface of each ureter at the sigmoid mesocolon root. Next, along the pelvic wall, the surroundings of the rectum were anatomized toward the anus. To clear expose the low structure of the rectum, 10 anatomical specimens were sagittally incised, forming 20 semi-pelvic cavities.

## Results

The UGF was found to be located on the deep side of the posterior peritoneum, with two types of layers, deep and superficial. The bilateral superficial layer mutually extended and ended in front of the abdominal aorta. At the posterior axillary line, the superficial layer medially reversed and continued to medially extend to a deep layer. The bilateral deep layer extended medially beside the vertebral body and then continued with the transversalis fascia. The kidneys were ensheathed by both layers. The ureters, genital vessels, and superior hypogastric plexus moved between both layers.

In the inguinal region, the deep and superficial layers ensheathed the vas deferens and spermatic vessels and moved through the deep inguinal ring. From the deep inguinal ring to the midline, the superficial layer extended to the posterior wall of the urinary bladder, whereas the deep layer extended to the anterior wall of the urinary bladder. The deep and superficial layers wrapped around the urinary bladder and extended along the medial umbilical ligament to the umbilicus.

In the sacral promontory, the deep and superficial layers extended along the sacrum, forming the presacral fascia. The superficial layer, i.e., the superficial layer of the presacral fascia, formed the rectosacral fascia at the S4 sacral vertebra, whereas the deep layer, i.e., the deep layer of the presacral fascia, extended to the pelvic diaphragm, terminating at the levator ani muscle. On the rectum’s lateral side, the neurovascular bundles moved between both layers.

## Discussion

**Fig. 1:**
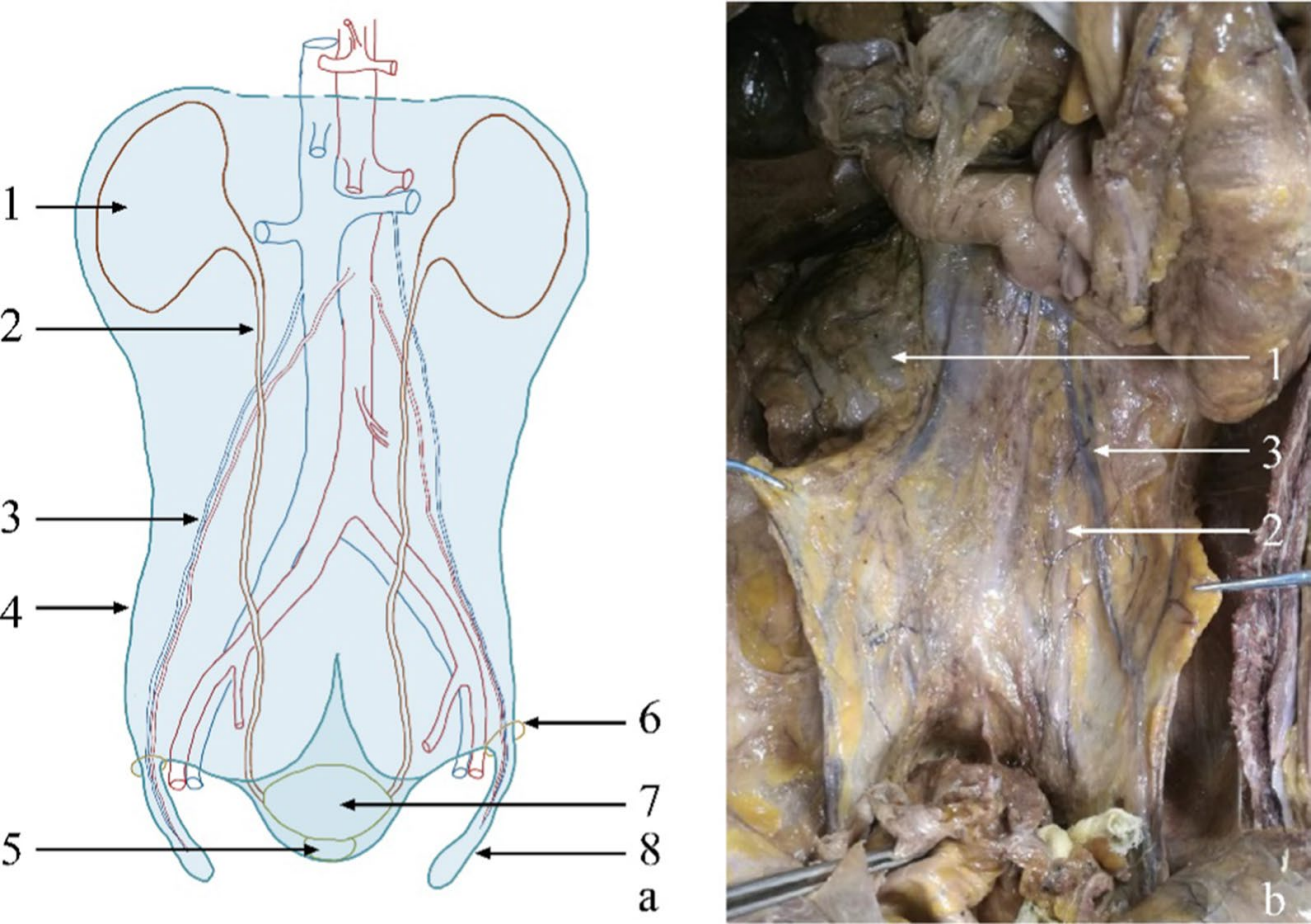
Overall appearance of the UGF. **a** Schematic diagram of the anatomical composition of the UGF. The light-blue shaded portion represents the UGF; the light-blue dotted line indicates the unclear boundary. **b** The partial entity of panel **a** in a cadaver. 1, kidney; 2, ureter; 3, genital vessels; 4, the lateral boundary of the UGF; 5, prostate; 6, deep inguinal ring; 7, urinary bladder; 8, spermatic sheath

Based on the anatomical results, it was found that a fascia system ensheaths the kidneys, ureters, vas deferens, genital vessels, superior hypogastric plexus, seminal vesicles, prostate, and urinary bladder. The fascia can be called “UGF,” which includes the deep UGF, superficial UGF, and the extension to the abdominal and pelvic wall (Fig. 1). To better understand the extension of the UGF, the superficial layer of UGF (towards the abdominal and pelvic organs) may also be called the visceral layer of UGF. Similarly, the deep layer of UGF (towards the body wall, pelvic wall) may also be termed the parietal layer of UGF. Additionally, the anterior renal, posterior renal, and presacral fasciae are defined as parts of the fascia system.

**Fig. 2:**
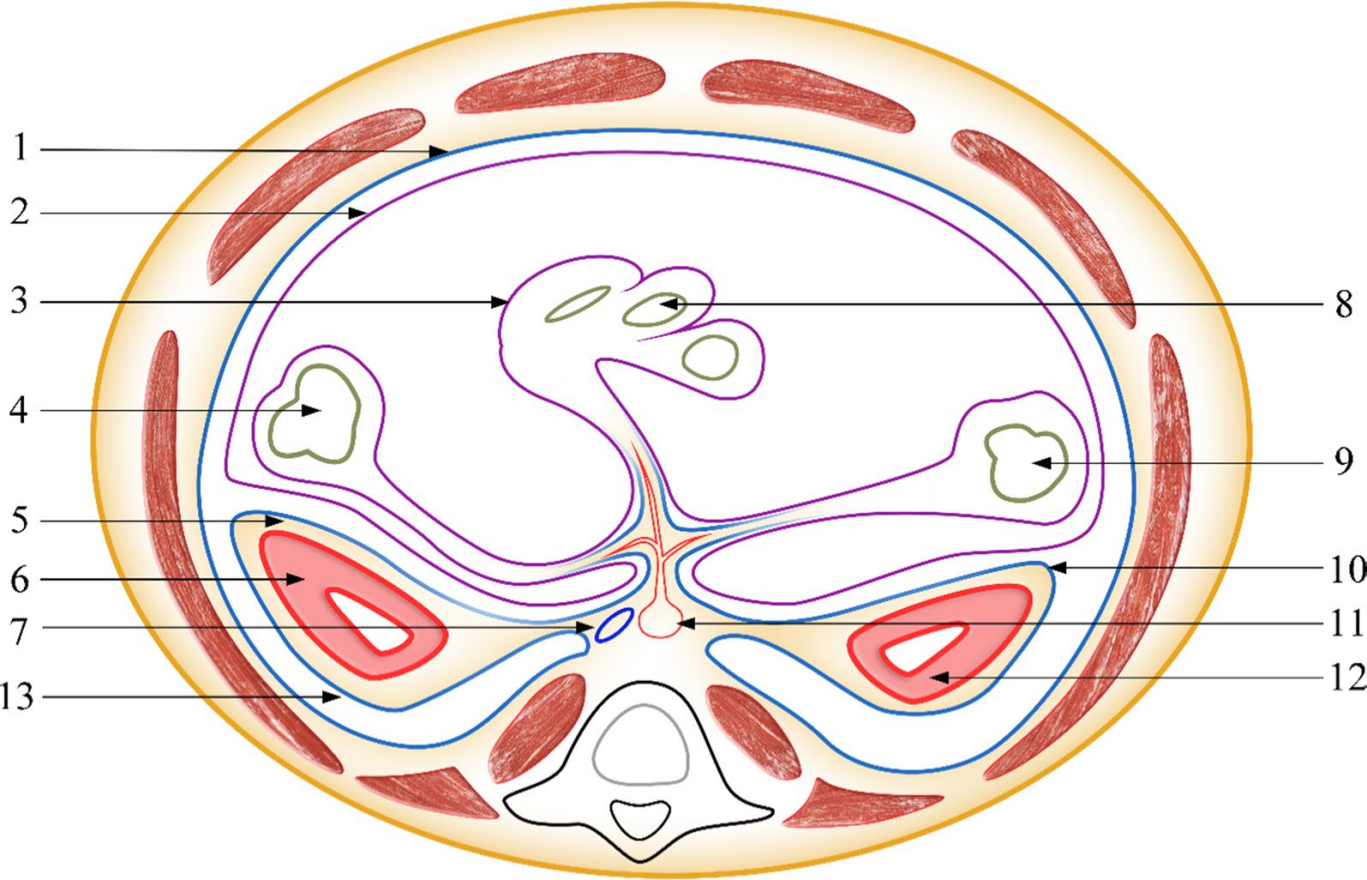
Cross-section of the UGF. 1, transversalis fascia; 2, parietal peritoneum; 3, visceral peritoneum; 4, ascending colon; 5, superficial layer of the UGF; 6, right kidney; 7, inferior vena cava; 8, small intestine; 9, descending colon; 10, the lateral boundary of the UGF; 11, abdominal aorta; 12, left kidney; 13, deep layer of the UGF

Many researchers have proposed the concept of the UGF, but descriptions of its boundaries are often confusing or even missing. The UGF has two layers, deep and superficial, in addition to a lateral and a bottom boundary. The lateral boundary of the UGF is the key to actually understanding the UGF. Kneeland [6] et al. and Thornton [7] et al. also confirmed that the bilateral superficial layer of the UGF mutually extends and ends in front of the abdominal aorta. Laterally, Mirilas [8] et al. believed that the deep and superficial layers of the UGF fuse on the posterolateral surface of the ascending or descending colon, forming the lateroconal fascia, which then merges with the transversalis fascia. However, anatomical studies have found that the superficial layer reverses laterally at the posterior axillary line and continues to medially extend to a deep layer. Therefore, the UGF forms a distinct lateral boundary (Fig. 2). Consistent with our results, Yang et al. [3] also found that the deep layer medially extends to communicate with the transversalis fascia. Raptopoulos et al. [9] believed that the deep layer medially continues with the lumbar muscle fascia. From the viewpoint of embryonic development, the genitourinary organs and pelvic wall muscle systems are derived from the mesoderm, and the UGF and transversalis fascia can be considered the “envelope” of the mesoderm’s inner layer. Therefore, the UGF and transversalis fascia should be homologous.

**Fig. 3:**
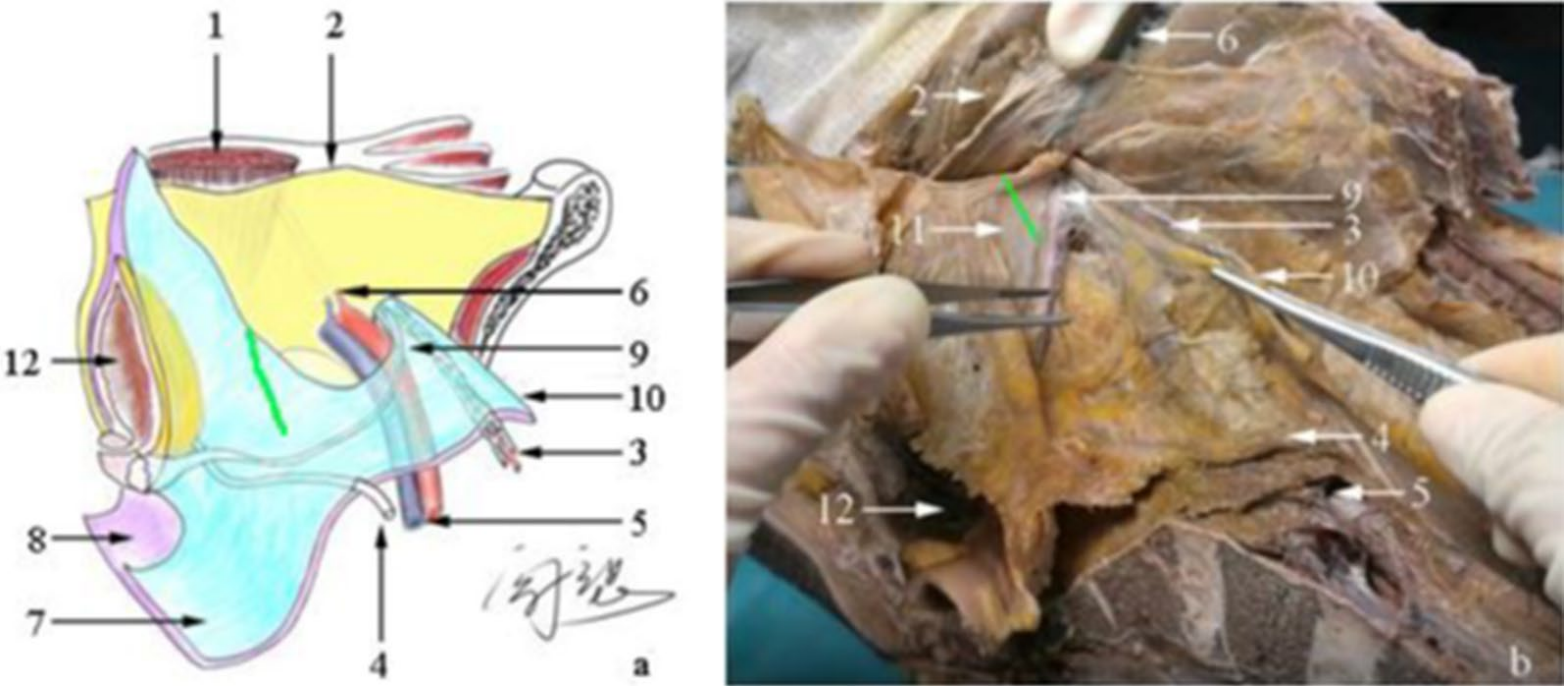
The urogenital fascia in the pelvis. **a** Schematic diagram of the UGF in the pelvis (view of the inner abdomen). **b** The partial entity of panel **a** in a cadaver. The UGF has a lateral boundary, laterally and inferiorly ensheathing the vas deferens and spermatic vessels and moving through the deep inguinal ring. Medially, the UGF is extended to the urinary bladder and pelvis. The ureters, vas deferens, and spermatic vessels move between both layers of the UGF. 1, rectus abdominis muscle; 2, transversalis fascia; 3, spermatic vessels; 4, ureter; 5, iliac vessels; 6, inferior epigastric vessels; 7, superficial layer of the UGF; 8, deep layer of the UGF; 9, vas deferens; 10, the lateral boundary of the UGF; 11, the transitional part of the UGF from the deep inguinal ring to the urinary bladder;12, urinary bladder. The green line indicates the transitional part of the UGF from the deep inguinal ring to the urinary bladder must be cut to flatten the mesh in LIHR

**Fig. 4:**
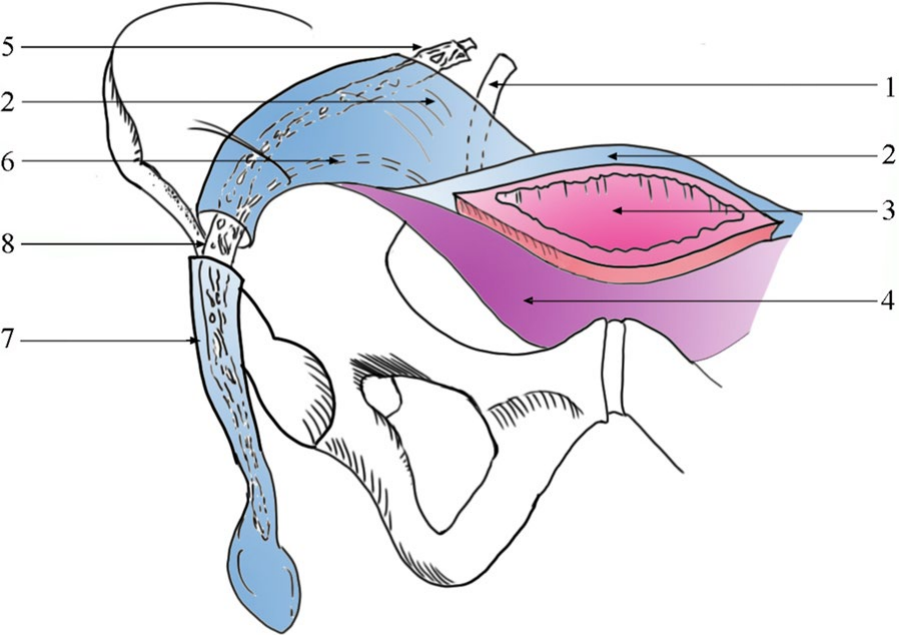
The UGF and the urinary bladder. 1, ureter; 2, superficial layer of the UGF; 3, urinary bladder; 4, deep layer of the UGF; 5, spermatic vessels; 6, vas deferens; 7, spermatic sheath; 8, spermatic cord. Laterally and inferiorly, the deep and superficial layers of the UGF extend to the spermatic sheath and move through the inguinal ring. Medially, the UGF ensheaths the urinary bladder. The superficial layer ensheaths the posterior urinary bladder wall and the deep layer ensheaths the anterior urinary bladder wall. The ureters, vas deferens, and spermatic vessels move between both layers.

In our study, it was found that the UGF coursed through the deep inguinal ring (Fig. 3). Stoppa et al. [10] also found a fascial system that laterally extends and ensheaths the vas deferens and spermatic vessels, forming the spermatic sheath, which is considered part of the UGF. However, the authors did not describe the extension from the deep and superficial layers of the UGF to the urinary bladder [10] (Fig. 4). The previous study [11] has reported that the preperitoneal fascia also surrounds the vas deferens and spermatic vessels.

According to our anatomy, the preperitoneal fascia and extraperitoneal fascia are part of the UGF. Sato [12] and Fowler [11] reported that the preperitoneal fascia has two layers. According to Sato, the preperitoneal fascia is a continuation of the renal fascia, which means a continuation of the UGF [12]. Fowler observed that the superficial layer of the preperitoneal fascia ensheaths the vas deferens and enters the deep inguinal ring, in addition to blending with the transversalis and internal spermatic fasciae [11]. Similarly, Arregui et al. [13] observed that the preperitoneal fascia ensheaths the hernial sac and spermatic cord. Besides, Lampe [14] suggested that the extraperitoneal fascia includes an outer layer and a fat layer. These results show that the preperitoneal and extraperitoneal fasciae are part of the UGF. However, these authors observed only local anatomical characteristics of the fasciae without focusing on their origin and boundary; therefore, the results had some deviations and deficiencies.

A clear understanding of the anatomy of the UGF, which medially extends from the deep inguinal ring to ensheath the urinary bladder, provides an anatomical basis for further understanding the relevant fascia of the urinary bladder and also helps identify various anatomical levels during surgery. The UPF extends from the umbilicus to the urinary bladder, which is located deep in the transversalis fascia [8]. The deep and superficial layers of the UGF extended to the anterior and posterior walls, respectively, of the urinary bladder. Therefore, the UPF described by Diarra et al. [1] should represent the deep layer of the UGF, whereas the umbilical vesical fascia described by Mirilas et al. [8] should be the intrinsic fascia of the urinary bladder. These results indicate that the intrinsic fascia of the urinary bladder is surrounded by the deep and superficial layers of the UGF, which extended from the urinary bladder to the umbilicus along the medial umbilical ligament. Mirilas et al. [8] also observed that the UPF extends from the umbilicus to the urinary bladder. Caudally, the UGF is continuously extended to form the presacral fascia, which is closely related to the lateral ligament of the rectum and the neurovascular bundle.

The confusion related to the anatomy of the inguinal region membrane led to controversy surrounding the transversalis fascia stratification, causing a difference in the understanding of the Retzius’ space. Although most researchers believe that the transveralis fascia has two layers, Read et al. [5] reported that the deep layer of the transversalis fascia is the superficial layer of the extraperitoneal fascia. We believe that the deep layer of the transversalis fascia is the deep layer of the UGF. According to the relationship among the UGF, urinary bladder, and transversalis fascia, when we performed LIHR, the Retzius’ space should be separated between the transversalis fascia and the deep layer of the UGF (namely, the deep layer of the transversalis fascia), whereas the Bogros’ space should be separated between the peritoneum and the superficial layer of the UGF. Separating the Retzius’ space between the deep and superficial layers of the UGF might damage the blood vessels on the surface of the urinary bladder, leading to uncontrolled bleeding. Similarly, peeling off the transversalis fascia might cause the corona mortis to become naked, leading to uncontrolled bleeding.

During LIHR, the interior side of the mesh should be placed between the transversalis fascia and the deep layer of the UGF, whereas its lateral side should be placed between the peritoneum and the superficial layer of the UGF. To flatten the mesh, the transitional part of the UGF from the deep inguinal ring to the urinary bladder must be cut (Fig. 3). Similarly, Nagahisa et al. [15] suggested that this part of the fascia should be incised. Our view of space separation and mesh placement is consistent with that of Mirilas et al. [16]. Also, the UGF on the surface of the vas deferens and spermatic vessels must be protected to reduce the adhesion between the mesh and vas deferens. The previous studies have summarized the complications associated with the mesh, such as mesh migration [17], mesh erosion into the urinary bladder [18], and visceral injury [19]. Proper mesh placement is key to preventing these complications. Knowledge of the anatomy of the UGF provides an anatomical basis for the correct determination of space and accurate placement of the mesh during LIHR. The application of the UGF in colorectal surgery requires further study.

## Conclusion

An independent UGF system with a deep and superficial layer exists in the body. The bilateral superficial layer of the UGF mutually extends and ends in front of the abdominal aorta, while the deep layer of the UGF continues with the transversalis fascia, beside the vertebral body. Superiorly, the two layers ensheath each kidney, while laterally ensheathing the ureters and genital vessels. Meanwhile, the partial UGF moves through the deep inguinal ring along with the vas deferens and spermatic vessels, forming a distinct boundary from the lateral border of each kidney to the deep inguinal ring. Next, from the deep inguinal ring to the midline, the deep and superficial layers ensheath the urinary bladder and extend along the medial umbilical ligament to the umbilicus. Inferiorly, the deep and superficial layers extend to the pelvic diaphragm. The superior hypogastric plexus, seminal vesicles, prostate, and neurovascular bundles move between the two layers of the UGF. Studying the UGF provides a comprehensive anatomical basis for understanding the membrane anatomy of the inguinal region, helping us unify the anatomical concepts of the preperitoneal fascia, the extraperitoneal fascia, and the UPF. Besides, it helps find the right anatomical level during LIHR, thereby reducing bleeding and other complications.

## Data Availability

The datasets generated and analyzed during the current study are available from the corresponding author on reasonable request.
